# 3D Printing of BaTiO_3_ Piezoelectric Ceramics for a Focused Ultrasonic Array

**DOI:** 10.3390/s19194078

**Published:** 2019-09-20

**Authors:** Jian Cheng, Yan Chen, Jun-Wei Wu, Xuan-Rong Ji, Shang-Hua Wu

**Affiliations:** School of Electromechanical Engineering, Guangdong University of Technology, Guangzhou 510006, China; jiancheng0372@foxmail.com (J.C.); wujunwei@cndoppler.cn (J.-W.W.); xr.ji@gdut.edu.cn (X.-R.J.); swu@gdut.edu.cn (S.-H.W.)

**Keywords:** stereolithography, BTO, piezoelectric ceramic, ultrasonic transducer

## Abstract

BaTiO_3_ (BTO) ceramics were fabricated based on stereolithography technology. The microstructures and electric properties of the BTO ceramics were studied. X-ray patterns of sintered BTO ceramics indicated that the tetragonal phase had formed, and the grain size increased clearly as BTO weight percentage increased. Moreover, the BTO ceramics exhibited good electric properties, with a piezoelectric constant d_33_ of 166 pC/N at 80% BTO weight percentage. To evaluate the properties of 3D printed BTO ceramics, a 1.4 MHz focused ultrasonic array was fabricated and characterized. The −6dB bandwidth of the array was 40%, and the insertion loss at the center frequency was 50 dB. The results show that the printed BTO ceramics array have good potential to be used in ultrasonic transducers for various applications.

## 1. Introduction

Transducer elements play a critical role on transducer performance, so proper piezoelectric material selection in ultrasonic transducer design is of utmost importance. Because the application of toxic lead-based materials, such as lead zirconate titanate (PZT) and (1−x)Pb(Mg_1/3_Nb_2/3_)O_3_-xPbTiO_3_ (PMN-PT), causes serious environmental concerns, there is an urgent demand for developing lead-free substitutes for ultrasonic imaging applications. BaTiO_3_ (BTO), has been widely studied, due to its high dielectric constant, good piezoelectric coefficient, and favorable electromechanical coupling coefficient [1,2,3,4].

Ultrasonic transducers with special-shape elements and distribution are urgently needed for novel ultrasonic technology and developing new directions [5], such as brain stimulation and imaging [6,7]. However, piezoelectric ceramics with complex shapes and structures are extremely difficult to achieve by traditional manufacturing techniques, due to their brittleness. In addition, traditional machining processes, such as dicing or laser cutting, may cause strength damage and depolarization of the piezoelectric ceramics, which could deteriorate the performance of electronic devices. The additive manufacturing process, also known as 3D printing, is a rapid prototyping process having the advantages of unlimited design flexibility and low production consumption, which has attracted considerable attention in fabricating special-shape ceramics [8,9]. Kim et al. from the University of California fabricated piezoelectric composites by digital projection printing [2]. Cui et al. at Virginia Tech printed piezoelectric materials with designed anisotropy and directional response, based on functionalized PZT nanoparticle colloids [10].

A stereolithography apparatus (SLA) is a form of 3D printing technology which usually uses ultraviolet light to cure photosensitive resin. It doesn’t require high energy laser beams to avoid the defects caused by internal stress. The ceramic-based components printed by SLA have high precision, as well as good surface quality [11,12]. There are some piezoelectric ceramics prepared using the SLA method, such as PZT [13,14,15,16], Pb(Mg_1/3_Nb_2/3_)-PbTiO_3_(PMN-PT) [17], K_0.48_Na_0.52_NbO_3_(KNN) [18], and BTO [19,20]. Our group printed PZT ceramics and fabricated a two-dimensional ultrasound array [16]. As for research about lead-free BTO printing, Jang et al. at Alfred University have prepared BTO UV-curable suspensions and characterized their rheological properties for stereolithography [19]. Chen et al. at the University of Southern California reported that BaTiO_3_ piezoelectric ceramics were printed for fabricating single-element focusing ultrasonic transducers and four-element annular piezoelectric arrays [20,21]. The weight percentages and microstructures of the printed BTO ceramics are essential for their properties’ enhancement. However, research on the relationships between weight percentage, microstructure, and the electric properties of lead-free BTO ceramics are lacking. To better achieve high-quality BTO ceramics for device applications, the effect of weight percentage on microstructures, piezoelectric, ferroelectric, and dielectric properties were investigated for this paper. Finally, a focused ultrasonic array was fabricated to further evaluate the properties of BTO ceramics.

## 2. Experimental

### 2.1. BTO Slurry and Green Parts

BTO powders (500 nm) with different weight percentages (70 wt%, 75 wt%, 80 wt%, 82 wt%, 84 wt%, and 86 wt%) and photosensitive resin were mixed by ball-milling. The photosensitive resins were prepared with moderate amounts of the oligomers, monomer, and UV-initiating agent. In order to obtain a homogeneous slurry, dispersant was also added to break up the aggregate. Then, the BTO slurry was spread out on the resin container and then exposured by the UV light in the SLA equipment. Green parts, with a disk size of φ12 × 3 mm, were printed for property characterization. Then, the debinding processes of the green parts included two steps, which were carried out to obtain dense ceramics [11]. Finally, the green parts were sintered at 1290 °C for 2 h.

### 2.2. BTO Ceramics Characterization

The microstructures of the BTO ceramics were observed using scanning electron microscopy (SEM, LYRA 3 XMU, Tescan, Brno, CZ). The density of sintered samples was measured by the Archimedes method. An X-ray diffractometer (XRD, X’Pert PRO, PANalytical B.V., Almelo, Netherlands) was used to determine the crystal structure of the printed samples. The BTO ceramics with silver electrodes were poled under an electric field of 30 kV/cm for 15 min in 70 °C silicone oil using poling equipment (Rek RK2674A, HYJH-3YY/20 kV). The piezoelectric constant d_33_ was determined by a piezo-d_33_ meter (ZJ-3A, Institute of Acoustics, Chinese Academy of Sciences, Beijing, China). The room temperature dielectric constant and electromechanical coupling coefficients of the ceramics were measured using an impedance analyzer (Agilent 4294A, Agilent Technologies, Santa Clara, CA, USA). The temperature-dependent dielectric properties of the BTO ceramics were also characterized by an impedance analyzer (TH2816, Tonghui, Changzhou, China). The polarization-electric field (P–E) hysteresis loops were acquired using a ferroelectric tester (Multiferroics, Radiant Technology, Albuquerque, USA) at room temperature. The focused ultrasonic array was characterized via a traditional method based on pulse-echo response measurement.

## 3. Results and Discussions

### 3.1. BTO Ceramics Characterization

#### 3.1.1. Microstructure 

**Figure 1:**
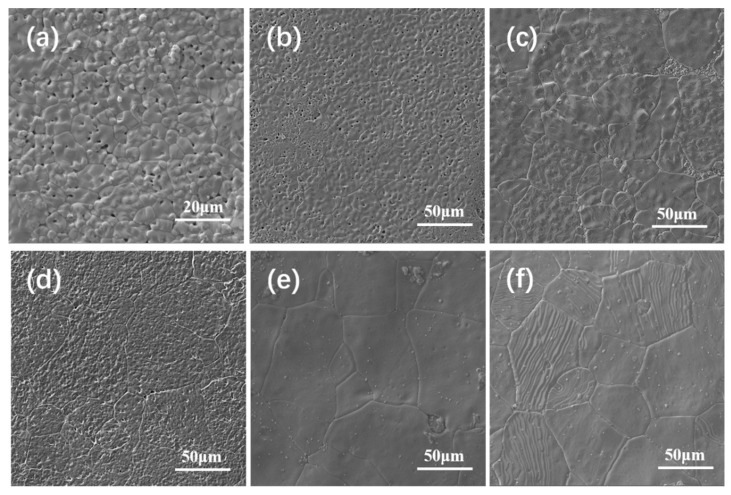
SEM micrographs of 3D-printed BaTiO_3_ (BTO) ceramics (**a**) 70 wt%; (**b**) 75 wt%; (**c**) 80 wt%; (**d**) 82 wt%; (**e**) 84 wt%; (**f**) 86 wt%.

The SEM micrographs of 3D-printed BTO ceramics with different weight percentages are given in Figure 1. It can be seen that the grain morphology is inhomogeneous with grain size in the range of 10–15 μm, and a large amount of pore appears at relatively low BTO weight percentage (70 wt%). With a BTO weight percentage increase, the grain size progressively increases, and the pore decreases and almost disappears when the amount of BTO exceeds 80%. The results show that the microstructures of the ceramics are sensitive to BTO weight percentage. The density of sintered BTO ceramics is shown in Table 1. It can be seen that density increases with the increase of BTO weight percentage and relatively high density is obtained as the BTO weight percentage becomes larger than 80%. It is well known that the BTO ceramics with higher BTO weight percentage have a larger density. However, as BTO weight percentage increases up to 82%, a large amount of dispersant is added to adjust the slurry viscosity for better 3D printing, hence leading to a slightly reduction in density.

#### 3.1.2. XRD Patterns 

**Figure 2:**
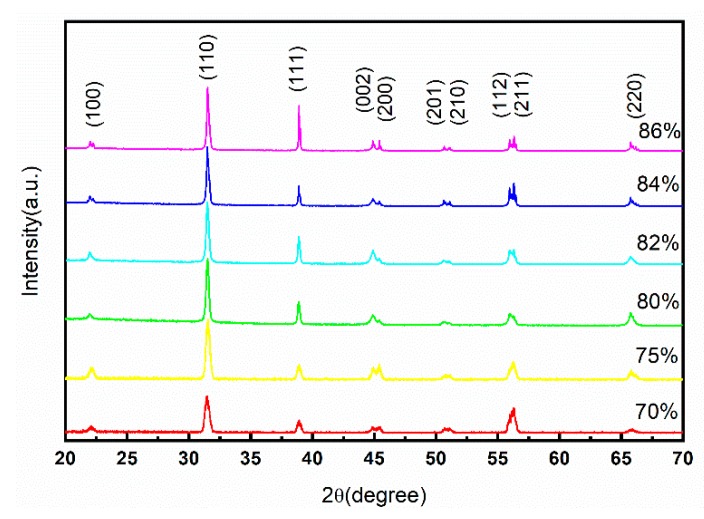
XRD patterns of BaTiO_3_ ceramics with different weight percentages.

Figure 2 shows the XRD patterns of 3D-printed ceramics with different BTO weight percentages. It can be seen that all BTO ceramics show a tetragonal perovskite structure, the intensity of XRD patterns increase, and the diffraction peaks become sharp as the BTO weight percentage increases, which indicates that good crystallinity can be formed and may also be affected by the increased grain size.

#### 3.1.3. Electrical Properties 

In order to characterize the properties of the 3D-printed BTO ceramics, ceramic disks (diameter~10 mm, thickness~0.6 mm) were prepared using the above fabrication process. The electrical properties of the BTO ceramics with different weight percentages are shown in Table 2. The piezoelectric constant d_33_ increases from 96 to 166 pC/N, almost reaching the maximum value, as the BTO weight percentage increases to 80%, and then decreases a little. The weak properties with relatively low BTO weight percentage (≤75%) may be related to the low density affected by the pores. Dielectric properties also showed the same phenomena for the samples with lower BTO weight percentages (≤75%). The electromechanical coefficient *k_t_* is in the range of 0.173–0.416. The enhancement of the electrical properties may be related to improved density and increased grain size as the BTO weight percentage increases. The reasons for the decrease of the piezoelectric constant after 80 wt% can be explained by the effects of more dispersant additions.

**Figure 3:**
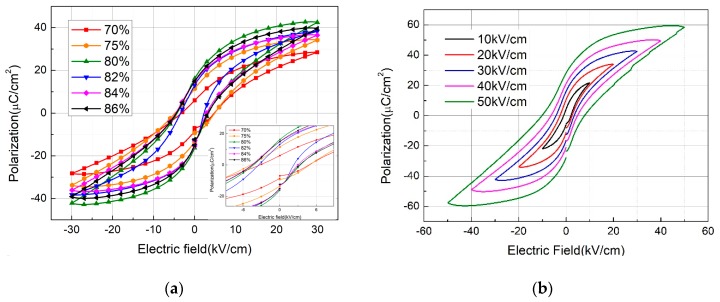
Polarization-electric field hysteresis loops of the printed ceramics with (**a**) different BTO weight percentages under 30 kV/cm (inset is the magnified one), (**b**) 80% BTO weight percentage under different electric fields.

To investigate the effects of BTO weight percentage on ferroelectric properties, the room temperature ferroelectric hysteresis loop of the printed samples, with different BTO weight percentages, was measured under an electric field of 30 kV/cm at 10 Hz (Figure 3a). Both the remnant polarization and coercive field increase as the BTO weight percentage increases to 80%, then remains almost unchanged. The increased remnant polarization may be explained by the decrease of barrier height for the ceramics, which is affected by the increased gain size and high density. The enlarged coercive field indicates that more energy is needed for the movement and reorientation of domain walls. Moreover, for the sake of better understanding the ferroelectric properties of the 80% BTO sample, the polarization-electric field(P-E) loops under different electric fields are shown in Figure 3b. The P-E loops become saturated as the electric field increases and the remnant polarization and coercive fields increase to 28.5 μC/cm^2^ and 10 kV/cm, respectively. The ferroelectric properties of the printed samples are comparable to that of BTO ceramics fabricated by using a traditional pressing method [22].

**Figure 4:**
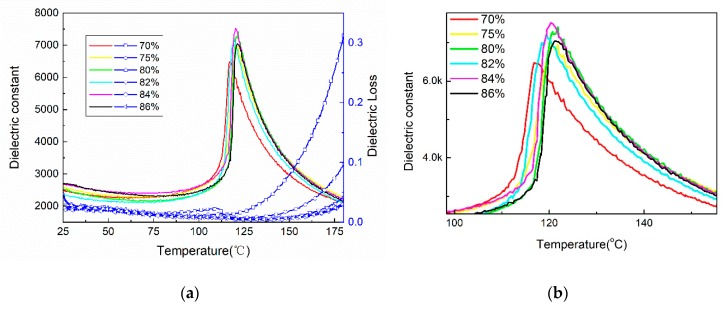
The temperature dependence of the dielectric constant and (**a**) dielectric loss, and the (**b**) magnified figure around Curie temperature.

The temperature dependences of the dielectric constant and dielectric loss at 1 kHz for printed BTO ceramics are shown in Figure 4. All printed samples show only one dielectric peak at about 120 °C, which corresponds to the Curie temperature. It can clearly be seen that the Curie temperature increases slightly as BTO weight percentage increases (Figure 4b), which may also be related to the increase of grain size, contributing to growth of the domains. The dielectric loss of all samples is smaller than 0.05 from room temperature to 130 °C. In summary, the 80% BTO weight percentage with relatively good properties was selected to fabricate the focused ultrasonic array.

### 3.2. 3D Printing Focused Ultrasonic Transducer

**Figure 5:**
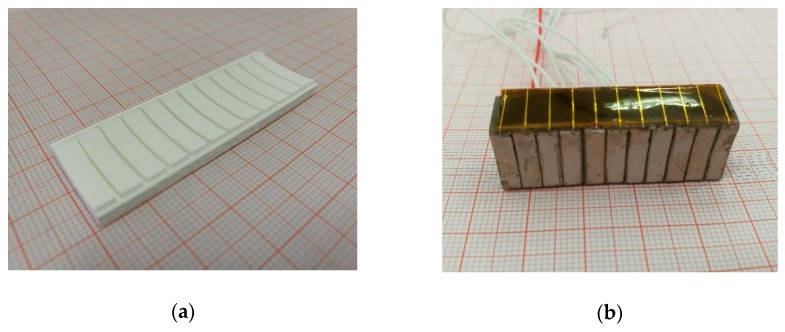
The photos of the 3D-printed (**a**) ceramic and (**b**) ultrasonic arrays.

**Figure 6:**
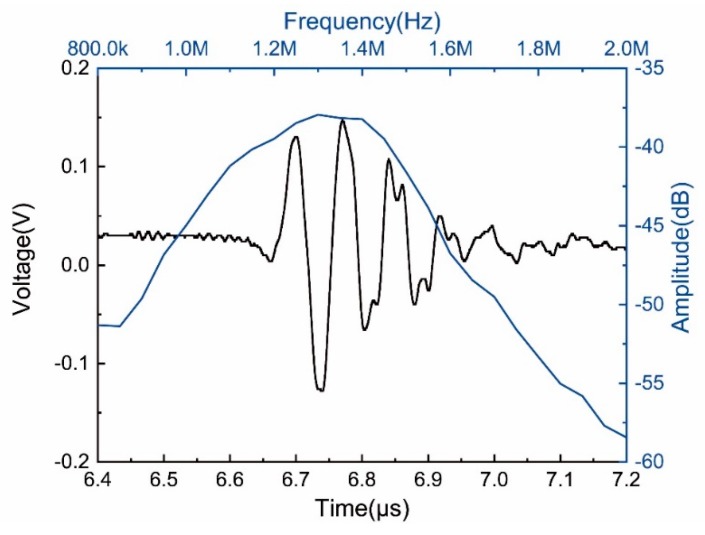
The pulse-echo waveform and frequency spectra of the focused array.

Here, a focused array was designed and fabricated with 80% BTO ceramics based on the SLA method. Figure 5 shows the photos of the 3D-printed BTO ceramic array and transducers. The ceramic array was printed with a curvature radius of 20.3 mm and thickness of 1.8 mm. The pitch and kerf are 4.5 mm and 0.5 mm, respectively. The prototype of the focused ultrasonic array is composed of BTO ceramics (as the active element), a backing layer, and cables. The backing layer, with a thickness of 10 mm, is casted onto the ceramic using a low-viscosity epoxy (Epo-Tek 301, Epoxy Technology, Billerica, MA, USA) mixed with tungsten powder and microbubbles. The acoustic impedance of the backing layer is about 5 MRalys. The kerfs between each element are filled with epoxy to reduce crosstalk. The tested array is placed in a water tank and towards a quartz target. An ultrasound pulser/receiver (JSR Ultrasonics DPR500, Pittsford, NY, USA), as well as an oscilloscope (Keysight DSOS054A), are connected to the element for characterization. Figure 6 shows the tested pulse-echo waveform and frequency spectra of the focused array. A~1.4 MHz focused array shows a wide bandwidth of 40% at −6 dB, and its insertion loss is about 50 dB, which exhibits promising performance when compared to the traditional PZT array [23]. The above results suggest that the 3D-printed BTO ceramics have good application prospects for complex-structure ultrasonic arrays.

## 4. Conclusions

3D-printed BTO ceramics with different BTO weight percentages (70%-86%) were prepared using stereolithography. The piezoelectric, ferroelectric, and dielectric properties of the 3D-printed ceramics were studied. The electric properties of ceramics clearly increased and then reduced slightly at 80 wt% with the increase of BTO weight percentage. A focused ultrasonic array with good performance was fabricated using 80% BTO ceramics, which demonstrated that 3D-printed BTO ceramics have good application prospects for complex-shape ultrasonic arrays.

## Figures and Tables

**Table 1:** Density of sintered ceramics with different BaTiO_3_ (BTO) weight percentage.

BTO Weight Percentage	70 wt%	75 wt%	80 wt%	82 wt%	84 wt%	86 wt%
**Density (g/cm^3^)**	5.52	5.57	5.65	5.62	5.63	5.68

**Table 2:** The electrical properties of the 3D-printed BTO ceramics.

BTO Weight Percentage Electrical Properties	70 wt%	75 wt%	80 wt%	82 wt%	84 wt%	86 wt%	BTO Ceramic [22]
d_33_ (pC/N)	96	110	166	130	124	122	190
ε_r_ (1 kHz)	1829	2175	2177	2210	2251	2276	1700
tan δ	0.094	0.076	0.036	0.040	0.036	0.036	<0.1
k_t_	0.29	0.36	0.41	0.42	0.39	0.17	-
